# Carriage rate and methicillin resistance of *Staphylococcus aureus* in food handlers in Kars City, Turkey

**DOI:** 10.1186/s40064-016-2278-2

**Published:** 2016-05-12

**Authors:** Leyla Vatansever, Çiğdem Sezer, Nebahat Bilge

**Affiliations:** Department of Food Safety and Public Health, Faculty of Veterinary Medicine, Kafkas University, 36100 Kars, Turkey

**Keywords:** *S. aureus*, Food handlers, Methicillin resistance

## Abstract

The aim of this study was determine carriage rate and methicillin resistance of *Staphylococcus aureus* among food handlers. Samples were collected by swabbing the mouth, nasal cavity and hands of food workers. The isolation of *S. aureus* was performed using a culture method and verified by using a genetic method (PCR). The presence of *mecA* gene was analysed by PCR. The fourteen antimicrobial disks were also used to determine antimicrobial susceptibility of *S. aureus*. The 56 out of 282 isolates were identified as *S. aureus*. It is found that 10 workers out of 28 carried *S. aureus* in their nasal cavity while 4 and 3 workers out of 21 carriered *S. aureus* in mouth and hands respectively. None of the isolates carried *mecA* genes and also their antibacterial susceptibility test for methicillin resistance, using cefoxitin (30 µg), shown that all the isolates was susceptible to methicillin. In conclusion, the results of the present study indicated that the prevalence of antibiotic resistance of *S. aureus* strains isolated from food handlers was low. However carriage rate of *S. aureus* among food handlers was quite high.

## Background

*Staphylococcus* is a genus of the Gram-positive bacteria and the member of the Staphylococcaceae family. In this genus the most common pathogen is *Staphylococcus aureus*, which having a broad host range. It is widely distributed in nature and is commonly found in the nose, throat, hair and skin of humans and animals. Approximately 20–30 % of healthy people carry this microorganism (Lory 2014; Van Belkum et al. 2009). However, *S. aureus* is a potentially dangerous pathogen causing a variety of localized or invasive diseases, as well as food poisoning.

Nasal carriage of *S. aureus* in food handlers is the major risk for staphylococcal food poisoning and because of a big concern for food safety. If the hygiene measures not taken up properly, organism can transferred from contaminated hands to food and can lead to food poisoning by the products of enterotoxigenic strains in this food (Ho et al. 2014). Both epidemiologic and molecular data in food poisoning outbreaks have indicated that *S. aureus* carried by food handlers was the source of contamination (Kluytmans et al. 1995; Sivaraman et al. 2009). Therefore it is important to detect *S. aureus* carriage among food handlers to prevent possible food contamination by them resulting in food poisoning.

Antimicrobial resistance is an inevitable consequence of antimicrobial use. Specifically the use of antimicrobials in food animals creates an important source of resistant bacteria. These bacteria can easily be spread to humans through the food supply (Collignon et al. 2009). *Staphylococcus aureus* can adapt better than any other human pathogen to the antimicrobials. It has demonstrated a unique ability to quickly respond to each new antimicrobial, with the development of a resistance mechanism, starting with penicillin and methicillin (Pantosti et al. 2007). Among *S. aureus*, methicillin-resistant strain (MRSA) has emerged as a serious life threaten infective agent which does not respond to a lot of antimicrobial treatments. This methicillin resistance strain synthesizes a penicillin binding protein (PBP2a), encoded by the *mecA* gene on a mobile genetic element, which has a role of counteracting the inhibitory effect of Beta-lactam antibiotics by preventing them from effectively binding to cell wall proteins (Pantosti et al. 2007).

In the present study, we aimed to determine carriage rate of *S. aureus* among food handlers work in catering services in Kars City, Turkey and to investigate their susceptibility to antibacterial agents using disk diffusion methods as well as methicillin resistance by using polymerase chain reaction.

## Methods

### Sample collection

The samples were collected from food handlers who work in catering services in Kars, Turkey. The catering services, 30 staff works in this establishment, prepares and delivers food to dining facilities of 2 academic institutions and some of them also work in services. Samples were obtained using sterile swabs and physiological saline solution from nasal cavities, hands and mouths of same food handlers, which work for service and preparation area. The workers deal with all kinds of meat (except pig meat) and vegetables. Three different swabs were used for one worker. One swab was used to swab areas in between fingers and the wrist area of the both hand and another swab was used to swab the 1–2 cm inside the anterior nares (one swab for both nares) and another swab used to swab mouth (internal face of cheeks). Sampling from the staff members was performed during their working hours on the same day. The experiment was conducted under the protocol which was approved by Kafkas University, Medicine Faculty, Local Ethical Committee (Decision No: 80576354-050-90/139).

### Isolation and identification of *Staphylococcus aureus*

Swab samples were streaked onto Baird-Parker Agar (BP, Oxoid CM 0275) with added Egg Yolk Tellurite (Oxoid, SR 0054). After incubation at 37 °C for 24–48 h, *S. aureus* suspected colonies (up to five colonies) were selected. The selected colonies were transferred into tubes containing Brain Heart Infusion Broth (BHI, Oxoid, CM375) and incubated at 37 ˚C for 24 h. Then transferred to BP agar incubated at 37 °C for 24–48 h and then transferred to Nutrient Agar slants (NA, Oxoid CM3) for further testing and then basic identification tests were performed on isolated colonies (ISO 1999; Bennett and Lancette 2001).

### Molecular typing of bacterial isolates

Genomic DNA was extracted from 24 h cultures in Brain Heart Infusion Broth (Oxoid CM 225) using a DNA extraction kit (Qiagen, 69506, DNeasy Blood and Tissue Kit, Germany) following the manufacturer’s instructions.

The each PCR mixture contained 100 ng genomic DNA, 1× taq buffer (Vivantis, S buffer with MgCl_2_ 17.5 mM), 1 U taq DNA polymerase (Vivantis, PL 1202-5/µl), 0.2 mM of dNTP mix (Vivantis, NP2406), 0.6 mM of forward and reverse primers (Sentromer/Turkey) and sterile Hypure water (HyClone-AUM57939) up to final volume of 25 µl. The sequence of the forward and reverse primer for *S. aureus* 5′-TGA AGT CAA ATA AAT CGC TT GC-3′ (Nuc2F) and 5′-CCC TTT TCC ACT AAT TCC TTA TT GT-3′ (NucR) (Perillo et al. 2012; Veras et al. 2008; Zhang et al. 2004).

The method suggested by Perillo et al. (2012) has been applied with some modifications in order to identify the *S. aureus* (*nuc*) genes. PCR was performed in a Biometra Thermocycler using the following conditions: 95 °C for 2 min (initial cycle), followed by 30 cycle of 95 °C for 30 s, 50 °C for 30 s and 72 °C for 30 s. ATCC 14458 and ATCC 43300 *S. aureus* strains were used as positive controls for PCR-based analysis. As negative control, Hypure water was used instead of template DNA. The PCR products were loaded onto a 2 % agarose gel containing ethidium bromide followed by electrophoresis using a 100 bp DNA ladder in TAE buffer at 90 V 60 min following visualization by an UV illuminator.

### Detection of methicillin resistance

The presence of the *mecA* gene was investigated by PCR. Sequences of the forward and reverse primers were: 5′-ACT GCT ATC CAC CCT CAA AC-3′ (GMECAR-1) and 5′-CTG GTG AAG TTG TAA TCT GG-3′ (GMECAR-2) (Mehrotra et al. 2000). The PCR parameters were an initial denaturation at 94 °C for 5 min, followed by 35 cycles of amplification at 94 °C for 2 min, 57 °C for 2 min, 72 °C for 1 min and 72 °C for 7 min (Mehrotra et al. 2000).

### Antimicrobial susceptibility

Antimicrobial susceptibility test of the *S. aureus* isolates was performed by agar disk diffusion method according to the Clinical and Laboratory Standards Institute (CLSI 2014) and European Committee on Antimicrobial Susceptibility Testing (EUCAST 2013). All tests were performed on Mueller–Hinton agar. The bacterial suspension having visually equivalent turbidity to 0.5 McFarland standards were prepared. The swab stick was dipped into bacterial suspension then took out and squeezed on the wall of the test tube to discard extra suspension. The surface of agar was then lightly and uniformly inoculated using this swab. Four antibiotic disks were placed for each plate. The plates were incubated at 35 °C for 24 h. After 24 h, inhibition zones on agar plate were measured. Results were recorded and graded as resistance (R) intermediate resistance (IR) and sensitive (S) according to the reference zone of inhibition of particular antibiotic (EUCAST 2015). *S. aureus* ATCC 25293 was used as a quality control standard. Following antibiotics were used susceptibility test: netilmicin (30 µg), gentamicin (10 µg), amikacin (30 µg), erythromycin (15 µg), imipenem (10 µg), ciprofloxacin (5 µg), cefoxitin (30 µg), tetracycline (30 µg), chloramphenicol (30 µg), cefazolin (30 µg), trimethoprim (5 µg), trimethoprim/sulfamethoxazole (25 µg), mupirocin (200 µg), clindamycin (2 µg).

## Results

A total of 282 *Staphylococcus* spp. isolates, 112 isolates from nasal cavity swabs, 92 isolates from hand and 78 isolates from mouth swabs were examined for *S. aureus* (*nuc*) genes (Table 1). The 56 (20 %) out of 282 isolates were identified as *S. aureus* which including 32 (57 %) isolates from the nasal cavity, 13 (23 %) isolates from mouth and 11 (20 %) isolates from hands of workers.

**Table 1:** Distribution of isolates according to the samples

Samples	Number of person	*Staphylococcus* spp.	*Staphylococcus aureus*
Nasal cavity	28	112	32
Hand	21	92	13
Mouth	21	78	11
Toplam	28^a^	282	56

^a^From same 21 person nasal cavity, hand and mouth samples were collected

It is found that 10 workers out of 28 carried *S. aureus* in their nasal cavity while 4 and 3 workers out of 21 carried *S. aureus* in mouth and hands respectively.

The results of antimicrobial resistance of *S. aureus* isolates were shown in Table 2. None of the isolates carried *mecA* genes and also their antibacterial susceptibility test for methicillin resistance, using cefoxitin (30 µg), showed that all the isolates was susceptible to methicillin.

**Table 2:** Antibacterial resistance of *S. aureus* isolates obtained from food handlers

Antibiotics	Mouth (n = 13)		Hand (n = 11)		Nose (n = 32)	
	R (%)	IR (%)	R (%)	IR (%)	R (%)	IR (%)
Netilmicin	–	–	–	–	–	–
Gentamicin	–	–	–	–	–	–
Amikacin	–	–	–	–	–	–
Erythromycin	1 (8)	–	–	–	–	–
Imipenem	–	–	–	–	–	–
Ciprofloxacin	–	–	–	–	–	–
Cefoxitin	–	–	–	–	–	–
Tetracycline	–	1 (8)	1 (9)	–	–	–
Chloramphenicol	–	–	–	–	–	–
Cephazolin	–	–	–	–	–	–
Trimethoprim	–	1 (8)	–	–	2 (6)	2 (6)
Trimethoprim/sulfamethoxazole	–	–	–	–	–	–
Mupirocin	–	2 (15)	–	–	–	–
Clindamicin	–	–	–	–	–	–

*R* resistance, *IR* intermediate resistance

## Discussion

Studies from different countries have indicated that food handlers, owing to their 20–30 % carriage rate, have been one of the major sources of food contamination with *S. aureus* (Lory 2014; Van Belkum et al. 2009). In this study 20 % of isolates were identified as *S. aureus* and the percentage rate were higher in nasal cavity isolates (57 %) than mouth (23 %) and hand (20 %) ones. Results showed that, nasal carriage rate among food handlers was 36 % (10 people out of 28), however mouth and hand carriage rate lower compered to nasal carriage rate, 19 and 14 % (4 and 3 people out of 21) respectively. Only in one worker, nasal cavity, mouth and hand samples found to be positive for *S aureus*. While one worker’s hand and nasal cavity samples shown positive results for *S. aureus*, one worker’s mouth and nasal cavity samples were positive for *S. aureus*. It is possible that the worker can contaminate or carries one strain from her/his nasal cavity to her/his mouth or/and hand or vice versa. Earlier study (Sezer et al. 2015) report that carriage rate of *S. aureus* among food handlers was 79 %. However, there are other studies indicating much lower prevalence among food handlers in Turkey. Nasal carriage rate of *S. aureus* among food workers in Turkey were determined as follows; 3 % in Antalya, 7 % in Kütahya, 15 % in Bursa, 15 % in Konya, 23 % in Şanlıurfa (Sepin-Özen et al. 2013). Nasal carriage rates of *S. aureus* show a large variation among studies, cities and countries. In a study done in Kuwait (Udo et al. 2009) the carriage rate was reported as 53 %, while in another investigation conducted in Hong Kong (Ho et al. 2014) it was 23 % among food handlers. Sivaraman et al. (2009) reviewed the extent and variability of nasal carriage rate and suggested that prevalence varies in industrialized nations from 26 to 35 % of the population, ethnicity and availability of medical care affect the carriage levels and developed nations have a higher percentage of carriage than the developing countries. In our study we found that a higher percentage of carriage rate than other studies done in Turkey. But, we looked at special group of people which work in catering services and they deal with food all the times. So it is possible that this food might contaminate them with these bacteria. Contradicted results may reflect variation in different populations living in different geographical regions and at the same time, workers work in different foods (meat, dairy products, vegetables and grains etc.). Supporting this theory Ho et al. (2014) reported that colonization rates were considerably higher in workers handling raw meats (30 %) than in non-exposed workers (13 %). Furthermore, differences in results could be based on various factors such as cleaning habits of workers, level of education, the hygiene of utensils-equipment and the environment the food handlers is working in and the regulations on inquest (Sivaraman et al. 2009; Ho et al. 2014; El-Shenawy et al. 2014). El-Shenawy et al. (2014) conducted cross sectional study of skin carriage (hands and face swabs) and found that 75 (38 %) of the 200 person were positive for the presence of *S. aureus*. Udo et al. (1999) identified 32 (18 %) *S. aureus* out of 174 isolates from the hands of healthy food handlers. They also found much higher results (92 %) from nasal cavity. Similarly, in our study, mouth and hand carriage rate much lower than nasal carriage rate among food handlers. Extended hygienic measures should be applied in food plants and restaurants to prevent food contamination with these bacteria by food handlers.

In the second part of our study, antimicrobial resistance profile of isolates has been investigated. The World Health Organisation has divided antimicrobials in three categories as critically important, highly important and important according to their importance in human medicine (Collignon et al. 2009). In this study, six critically important antimicrobials (namely; gentamicin, netilmicin, imipenem, amikacin, erythromycine, ciprofloxacin), six highly important antimicrobials (cefazolin, trimethoprim, trimethoprim/sulfamethoxazole, tetracycline and cefoxitin) and two important antimicrobials (clindamycin, mupirocin) were tested. One isolates found to be resistant to critically important antimicrobials and eight isolates were resistant to two highly important antimicrobials. Multi drug resistance (MDR) was determined from a mouth isolates, which was resistant to erythromycin and mupirocin but none of the isolates were methicillin resistant. Sepin-Özen et al. (2013) reported that percentage of MRSA as 5.3 among food handlers work in Antalya, Turkey. In other study, nasal carriage rate of MRSA reported as 18 % (6 out of 34 farm workers) among farm workers in Turkey (Hazımoğlu 2011). In a study conducted on food handlers it was reported that only one isolate out of 200 isolates (Udo et al. 2009) was methicillin resistant. Similarly, Kamal et al. (2013) also found that none of the hand swabs of *S. aureus* isolates from the dairy workers and food handlers was positive for the *mecA* gene. Collignon et al. (2009) reported that although it currently represents a low proportion of the total MRSA infections that occur in people, there are an increasing number of reports of animal-derived MRSA especially from pigs, causing community-onset infection in people.

## Conclusion

In conclusion, the results of the present study indicated that the prevalence of antibiotic resistance of *S. aureus* isolated from food handlers was low. However, the carriage rate of *S. aureus* among food handlers was quite high. Since research findings and investigations have suggested that staphylococcal food borne disease is largely due to faulty food handling practices, it is important to give proper training to food handlers in order to prevent the contamination of foods.
